# Oral Corticosteroids Dependence and Biologic Drugs in Severe Asthma: Myths or Facts? A Systematic Review of Real-World Evidence

**DOI:** 10.3390/ijms22137132

**Published:** 2021-07-01

**Authors:** Luigino Calzetta, Marina Aiello, Annalisa Frizzelli, Giuseppina Bertorelli, Paola Rogliani, Alfredo Chetta

**Affiliations:** 1 Respiratory Disease and Lung Function Unit, Department of Medicine and Surgery, University of Parma, 43126 Parma, Italy; marina.aiello@unipr.it (M.A.); annalisa.frizzelli@unipr.it (A.F.); giuseppina.bertorelli@unipr.it (G.B.); alfredoantonio.chetta@unipr.it (A.C.); 2 Unit of Respiratory Medicine, Department of Experimental Medicine, University of Rome “Tor Vergata”, 00133 Rome, Italy; paola.rogliani@uniroma2.it

**Keywords:** monoclonal antibody, OCS dependence, oral corticosteroid, real-world, severe asthma, systematic review

## Abstract

Airway inflammation represents an important characteristic in asthma, modulating airflow limitation and symptom control, and triggering the risk of asthma exacerbation. Thus, although corticosteroids represent the cornerstone for the treatment of asthma, severe patients may be dependent on oral corticosteroids (OCSs). Fortunately, the current humanised monoclonal antibodies (mAbs) benralizumab, dupilumab, mepolizumab, omalizumab, and reslizumab have been proven to induce an OCS-sparing effect in randomized controlled trials (RCTs), thus overcoming the problem of OCS dependence in severe asthma. Nevertheless, a large discrepancy has been recognized between selected patients enrolled in RCTs and non-selected asthmatic populations in real-world settings. It is not possible to exclude that the OCS-sparing effect of mAbs resulting from the RCTs could be different than the real effect resulting in clinical practice. Therefore, we performed a systematic review and correlation analysis to assess whether mAbs are effective in eliciting an OCS-sparing effect and overcoming the OCS dependence in severe asthmatic patients in real-world settings. Overall, real-world studies support the evidence that OCS dependence is a real condition that, however, can be found only in a small number of really severe asthmatic patients. In most patients, the dependence on OCS can be related to modifying factors that, when adequately modulated, may lead to a significant reduction or suspension of OCS maintenance. Conversely, in severe asthmatics in whom OCS resistance is proved by a high daily dose intake, mAbs allow reversion of the OCS dependence, leading to the suspension of OCS therapy in most patients or >50% reduction in the daily OCS dose.

## 1. Introduction

The current recommendations from the global strategy for asthma management and prevention (GINA 2021) [1] define asthma as a heterogeneous disease characterized by chronic airway inflammation; asthmatic patients have a history of respiratory symptoms (wheeze, shortness of breath, chest tightness, cough) that may vary in intensity and over time, along with variable airflow limitation. Certainly, airway inflammation represents an important and treatable characteristic in asthma, and the level of airway inflammation may modulate airflow limitation and symptom control, and trigger the risk of asthma exacerbation [2,3]. Therefore, anti-inflammatory drugs, and specifically corticosteroids, represent the cornerstone for the treatment of asthma, although this class of drugs is characterized by dose-dependent adverse events and drug dependence [4].

While in most asthmatic patients inhaled corticosteroids (ICSs) work fine, in patients suffering from severe asthma, there is a possibility of dependence on oral corticosteroids (OCSs), a condition leading to poor disease control and high risk of exacerbation despite high-dose OCSs [4]. Generally, asthmatic patients with type 2-low inflammation are resistant to OCSs, whereas most patients with persistent eosinophilic inflammation respond to OCSs [5]. Although severe asthmatic patients with OCS dependence are a small proportion of the general asthmatic population, they represent a large burden on health care costs, with an important increase in morbidity, hospitalization, and mortality [6,7].

Several cellular and molecular mechanisms, inherited or acquired, can be related with corticosteroid resistance. It seems that genetic variations in up to 11 different genes can be associated with corticosteroid resistance in severe asthma [8]. The GLCCI1 gene, encoding the glucocorticoid-induced transcript 1 protein, has been extensively investigated and it is strongly associated with corticosteroid resistance in asthmatic patients [9,10,11]. Although many additional genes seem to have a significant relationship with corticosteroid resistance, the current evidence is conflicting across the studies [4]. Multiple acquired mechanisms have been proved to be related with corticosteroid resistance, namely the reduced glucocorticoid receptor (GR)-α expression, altered binding between GR and corticosteroids and defective binding between GR complex and DNA, GR antagonism due to enhanced transcription of pro-inflammatory factors or increased expression of GR-β, GR phosphorylation by p38 mitogen-activated protein kinase (MAPK), and reduced expression of anti-inflammatory genes induced by GC activation due to altered activity of histone deacetylase 2 (HDAC2), a condition often associated with smoking [12,13,14,15,16,17].

Fortunately, in the last two decades, a new class of biological treatments has been introduced to treat severe asthmatic patients. These biological agents are humanized monoclonal antibodies (mAbs) anti-IgE (omalizumab), anti-IL-5 (mepolizumab and reslizumab), anti-IL-5Rα (benralizumab), and anti-IL-4/IL-13 (dupilumab) [18,19,20,21]. All these mAbs have been extensively proved to induce a significant OCS-sparing effect in randomized controlled trials (RCTs), and thus overcome the problem related to OCS dependence in severe asthma [22,23,24,25,26].

However, since a large discrepancy has been extensively recognized between selected patients enrolled in RCTs and non-selected asthmatic populations in real-world settings [27,28], we cannot exclude that the OCS-sparing effect of the currently approved mAbs resulting from the RCTs could be different than the real effect resulting in clinical practice in severe asthmatic patients. To date, no systematic reviews on the OCS-sparing effect of mAbs have been performed in real-world settings. Therefore, the aim of this systematic review was to provide a synthesis of the current literature on the OCS-sparing effect of benralizumab, dupilumab, mepolizumab, omalizumab, and reslizumab in studies carried out in real-world populations of severe asthmatic patients and assess whether these mAbs may really overcome the problem related with dependence on OCSs in severe asthma.

## 2. Materials and Methods

### 2.1. Review Question

The question of this systematic review was to assess whether mAbs are effective in eliciting an OCS-sparing effect and overcoming the OCS dependence in severe asthmatic patients in real-world settings.

### 2.2. Search Strategy and Study Eligibility

**Figure 1:**
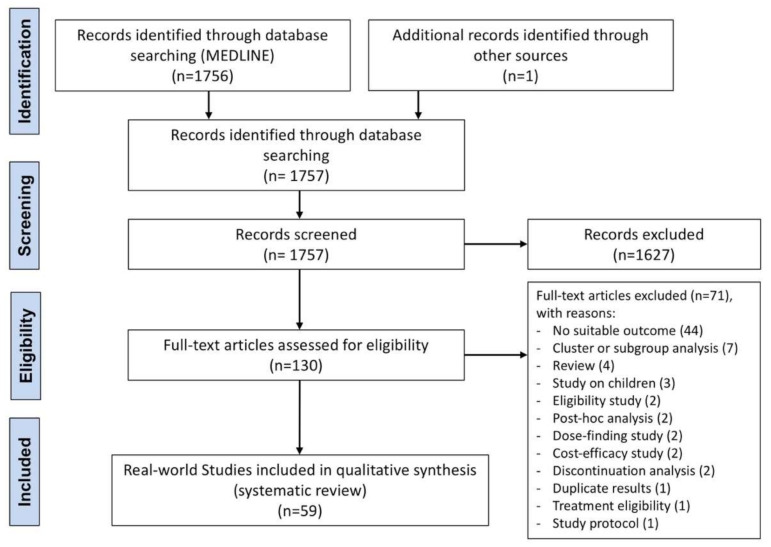
PRISMA flow diagram for the identification of the studies included in the systematic review. PRISMA, Preferred Reporting Items for Systematic Review and Meta-Analysis.

The protocol of this synthesis of the current literature was performed in agreement with the Preferred Reporting Items for Systematic Review and Meta-Analysis Protocols (PRISMA-P) [29], with the relative flow diagram shown in Figure 1. This study satisfied all the recommended items reported by the PRISMA-P checklist [29].

The PICO (Patient problem, Intervention, Comparison, and Outcome) framework was applied to develop the literature search strategy and question, as previously reported [30]. Namely, the “Patient problem” included adult severe asthmatic patients; the “Intervention” regarded the administration of mAbs; the “Comparison” was performed with respect to baseline; and the assessed “Outcome” was the use and dose of OCS.

The search was performed in MEDLINE in order to identify relevant studies available with no time limit up to 5 May 2021.

The research string was as follows: (“benralizumab”[Supplementary Concept] OR “benralizumab”[All Fields] OR (“dupilumab”[Supplementary Concept] OR “dupilumab”[All Fields]) OR (“mepolizumab”[Supplementary Concept] OR “mepolizumab”[All Fields]) OR (“omalizumab”[MeSH Terms] OR “omalizumab”[All Fields] OR “omalizumab s”[All Fields]) OR (“reslizumab”[Supplementary Concept] OR “reslizumab”[All Fields])) AND (“asthma”[MeSH Terms] OR “asthma”[All Fields] OR “asthmas”[All Fields] OR “asthma s”[All Fields]) AND (“difficult-to-treat”[All Fields] OR ((“difficult”[All Fields] OR “difficults”[All Fields]) AND (“therapy”[MeSH Subheading] OR “therapy”[All Fields] OR “treat”[All Fields] OR “therapeutics”[MeSH Terms] OR “therapeutics”[All Fields] OR “treating”[All Fields] OR “treated”[All Fields] OR “treats”[All Fields])) OR ((“difficult”[All Fields] OR “difficults”[All Fields]) AND (“controling”[All Fields] OR “controllability”[All Fields] OR “controllable”[All Fields] OR “controllably”[All Fields] OR “controller”[All Fields] OR “controller s”[All Fields] OR “controllers”[All Fields] OR “controlling”[All Fields] OR “controls”[All Fields] OR “prevention and control”[MeSH Subheading] OR (“prevention”[All Fields] AND “control”[All Fields]) OR “prevention and control”[All Fields] OR “control”[All Fields] OR “control groups”[MeSH Terms] OR (“control”[All Fields] AND “groups”[All Fields]) OR “control groups”[All Fields])) OR “difficult-to-control”[All Fields] OR (“refractories”[All Fields] OR “refractoriness”[All Fields] OR “refractory”[All Fields]) OR (“uncontrollability”[All Fields] OR “uncontrollable”[All Fields] OR “uncontrollably”[All Fields] OR “uncontrolled”[All Fields]) OR (“sever”[All Fields] OR “severe”[All Fields] OR “severed”[All Fields] OR “severely”[All Fields] OR “severer”[All Fields] OR “severes”[All Fields] OR “severing”[All Fields] OR “severities”[All Fields] OR “severity”[All Fields] OR “severs”[All Fields])).

Citations of previously published relevant reviews were examined to select further pertinent studies, if any [31]. Two reviewers independently checked the relevant studies identified from the literature search. The studies were selected in agreement with previously mentioned criteria and any difference in opinion about eligibility was resolved by discussion leading to consensus [32].

### 2.3. Data Extraction

Data from included studies were extracted in agreement with Data Extraction for Complex Meta-anALysis (DECiMAL) recommendations [33], and checked for study author, year, reference, mAbs, study characteristics, country, study duration, number of patients, disease characteristics, age of participants, sex, and dose of OCS at baseline. In the whole text, the daily dose of OCSs is reported as prednisone equivalent.

### 2.4. Endpoints

The endpoint of this systematic review was to assess the impact of mAbs on the use and dose of OCS in adult severe asthmatic patients.

### 2.5. Strategy for Data Analysis

Data from original papers were extracted and reported via qualitative synthesis. The correlation analyses between the reduction in the dose of OCS induced by mAbs and either the level of OCS dose at baseline, or the size of study population, or the study duration were carried out using the Pearson’s correlation analysis and graphically expressed via linear regression. The level of statistical significance was defined as *p* < 0.05.

## 3. Results

### 3.1. Study Characteristics

Of the 1757 potentially relevant records identified in the initial search, 59 real-world studies were deemed eligible for a qualitative analysis. Six studies were carried out on benralizumab [34,35,36,37,38,39], 1 on dupilumab [40], 18 on mepolizumab [41,42,43,44,45,46,47,48,49,50,51,52,53,54,55,56,57,58], 27 on omalizumab [18,59,60,61,62,63,64,65,66,67,68,69,70,71,72,73,74,75,76,77,78,79,80,81,82,83,84], and 3 on reslizumab [85,86,87]. Four studies investigated different mAbs in the same report [88,89,90,91]. The main characteristics of the real-world studies included in this systematic review are reported in Table 1.

### 3.2. Benralizumab

In a large multicenter study [34], 6 months of treatment with benralizumab completely abolished the OCS consumption in patients suffering from severe eosinophilic asthma, with the same decreases of daily OCS intake of −5 mg in patients with either positive or negative skin prick test. In another smaller study from the same authors [38] on patients suffering from atopic severe eosinophilic asthma, 6 months of treatment with benralizumab permitted OCS administration to be stopped in 81.8% of patients; in the remaining subjects, the OCS consumption was consistently reduced. After only 4 weeks of treatment, benralizumab completely abolished the use of OCS from 15.6 mg/day to 0 mg/day in patients with severe allergic eosinophilic asthma [39]. Accordingly, in a smaller study [35], all the OCS-dependent patients were able to discontinue such treatment after 3 months of benralizumab administration.

In severe refractory eosinophilic asthma, the dose of OCS at 3 months of benralizumab treatment decreased from 19.6 mg/day to 7.5 mg/day, and it continued to further decrease up to 6 months of treatment, at a level of 5 mg/day [37]. In another study [36] performed in severe refractory eosinophilic asthmatic patients, add-on therapy with benralizumab allowed complete suspension of OCS in 95% of patients and reduced the OCS dose from 18.7 mg/day to 0.25 mg/day after 6 months of treatment.

Overall, benralizumab elicited a rapid and significant OCS-sparing effect in severe asthmatic patients.

### 3.3. Dupillumab

Only one study [40] investigated the OCS-sparing effect of dupilumab in real world settings. After 1 year of treatment, dupilumab reduced from 20 mg/day to 5 mg/day the dose of OCS in patients with severe asthma. Twenty-four percent of patients completely suspended OCS, and 78% of patients reduced ≥50% the dose of OCS.

### 3.4. Mepolizumab

In very large studies [42,50] performed in patients suffering from severe eosinophilic asthma, 34% to 45% of patients discontinued OCS therapy after 1 year of treatment with mepolizumab. The study of Thomas et al. [42] on Australian national registry reported that the OCS dose reduced from 10 mg/day to 2 mg/day, and that the proportions of subjects receiving OCS bursts was reduced from 96% to 50%. The international study of Harrison et al. [50] showed that the OCS dose was reduced between 23% and 51% according to the blood eosinophil count. In another very large study from a commercial database in the USA [56], mepolizumab reduced by 14.7% the proportion of patients with a ≥1 OCS claim from baseline to follow-up (1 year). Mepolizumab also reduced the numbers of OCS claims/patient and OCS bursts by 29.1% and 36.8%, respectively. Reductions in OCS use were demonstrated during follow-up in patients with baseline mean OCS dose ≥5 mg. Furthermore, the proportion of patients who did not require OCS increased by 13.7% at follow-up.

Enríquez-Rodríguez et al. [41] reported that the number of patients requiring OCS treatment decreased to 19% in 1 year, and that the OCS dose was reduced from 18 mg/day to 9 mg/day. Similar data were reported also by Caminati et al. [45] during a shorter period of observation, after 6 months mepolizumab decreased the OCS dose by 5 mg/day, and the percentage of patients requiring OCS was reduced to 32.2%. These data were confirmed also by a study of Pertzov et al. [49], in which 68% of patients discontinued OCS treatment or reduced the daily dose >50% after 6 months of treatment with mepolizumab. Furthermore, the OCS dose was reduced from 20 mg/day to 5 mg/day.

In a large and long-term study [46] carried out in severe eosinophilic asthmatic patients, the treatment with mepolizumab for 2.5 years elucidated a significant and maintained reduction by 50% in the dose of oral corticosteroids as compared to baseline. This data was confirmed by another large and long-term study [47] in which, after 1 year and 2 years of treatment with mepolizumab, the use of OCS dropped from 92.8% at baseline to 41.1% and 34.7%, respectively. Moreover, patients still using OCS required lower doses (−62.1%) after 2 years of treatment. Interestingly, similar trends were seen when data were stratified by blood eosinophil counts at inclusion [47].

In a large study [44] performed in patients suffering from severe uncontrolled asthma, after 1 year of treatment with mepolizumab, only 28% of patients still required daily OCS, with a dose reduction from 10.1 mg/day to 2.0 mg/day. Analogously, another large study [57] showed that 1 year of treatment with mepolizumab permitted the number of severe asthmatic patients that needed OCS to be decreased by 45.4% and the OCS dose was reduced in 46.4% of patients.

In severe persistent eosinophilic asthma, the percentage of patients that received daily OCS dropped from 76% before starting mepolizumab treatment to 12% after 1 year of treatment [54]. Additionally, in the study of van Toor et al. [58], the percentage of patients requiring OCS was reduced to 15.4% after 1 year of treatment with mepolizumab. The predesigned interim analysis of a 2-year study [53] reported that in patients who completed 1 year of therapy with mepolizumab, a 56% reduction in the dose of OCS was achieved, from 10.1 mg/day to 4.5 mg/day. This interim analysis [53] also evidenced that, at the end of 1 year of therapy, 40% of patients completely suspended OCS.

Data from smaller studies are generally consistent with those from larger investigations. After 1 year of treatment with mepolizumab, only 16.1% of patients suffering from severe refractory eosinophilic asthma were still on OCS [52]. Pelaia et al. [48] reported that 24 weeks of treatment with mepolizumab decreased the intake of OCS from 24.11 mg/day to 1.78 mg/day, and that such a reduction persisted for many more weeks in those patients who were monitored for longer periods of time. In a small study [43] performed in severe eosinophilic asthmatic patients with chronic rhinosinusitis and nasal polyps, the dose of OCS was reduced in all patients after 24 weeks of treatment with mepolizumab, from 9.2 mg/day to 1.3 mg/day. OCS was discontinued in 40% of the study population. In patients suffering from severe eosinophilic asthma and inadequate asthma symptom control, mepolizumab reduced the OCS dose from 6.25 mg/day to 2.5 mg/day after 8 weeks [55]. Another small study [51] evidenced that the use of mepolizumab in severe eosinophilic asthma was associated with a decrease in mean daily OCS intake, and that most patients permanently discontinued OCS maintenance therapy after 3 months of therapy.

Overall, data from small to very large studies confirm that mepolizumab is effective in inducing a significant OCS-sparing effect in severe asthmatic patients, and that this effect is related to the level of the blood eosinophil count.

### 3.5. Omalizumab

Some real-world international studies have been carried out on omalizumab [74,77,80]. A very large study [74] on an international registry database showed that of the 49.8% of uncontrolled persistent allergic asthmatic patients receiving OCS at baseline, the proportion of those receiving OCS was reduced to 16.1% and 14.2% after 1 year and 2 years of treatment with omalizumab, respectively. The OCS dose decreased from 15.5 mg/day to 7.7 mg/day after 1 year and 5.8 mg/day after 2 years. Another very large international study [77] reported that administering omalizumab for >16 weeks permitted the use of OCS to be stopped in 20.5% of severe persistent allergic asthmatic patients and reduce the dose of OCS in 30.1% of subjects. Omalizumab allowed a reduction of the OCS dose by 74.3%, leading to an OCS reduction of 15.4 mg/day. Data from a large international registry database showed that in severe uncontrolled allergic asthmatic patients observed for 2 years, omalizumab decreased by 52.6% the proportion of subjects on OCS. The dose of OCS was reduced from 11.6 mg/day to 6.4 mg/day [80].

Several national real-world studies have been carried out on omalizumab [59,60,62,65,67,68,69,78,82,84]. In a very large and long-term national study performed in Poland [78] on severe asthmatic patients, the OCS dose decreased from 10.8 mg/day to 2.7 mg/day after 4 years of treatment with omalizumab, with a significant reduction observed at week 16, when 56% of patients had not used OCS at all. A very large national study [59] from a national pharmaceutical agency in Japan showed that treatment with omalizumab reduced the OCS dose of 10.4% at week 16 and 50.3% at week 52, when more than 80% of patients had a ≥50% OCS dose reduction and 13.3% had a ≥90% reduction. Another very large national registry study [65] performed in the Netherlands in inadequately controlled severe allergic asthmatic patients indicated that the percentage of patients that used OCS was reduced by 5.7% after 4 months of treatment with omalizumab. A large national database study [67] performed in Taiwan reported that in moderate to severe asthmatic patients, omalizumab reduced the use of OCS by 65.6%, 67.4%, and 72.3% after 2 months, 6 months, and 1 year of treatment, respectively. In a study on an Australian national registry database [68], after 6 months of treatment with omalizumab, 27.5% of severe allergic asthmatic patients with high prevalence of comorbidities reached a 25% reduction in the daily OCS dose. Data from a national registry from Portugal [69] reported that the percentage of uncontrolled allergic asthmatic patients on OCS was reduced from 17.7% at baseline to 9.3% and 8.2% after 1 and 2 years of treatment with omalizumab, respectively. Moreover, the maximum reduction in the dose of OCS was 5.3 mg/day. In a retrospective analysis [84] of electrical medical records, including Korean severe asthmatic patients, the dose of OCS was reduced by omalizumab from 4.4 mg/day to 3.4 mg/day. Another study [60] from USA electronic medical records performed in moderate to severe allergic asthmatic patients indicated that 1 year of treatment with omalizumab reduced the likelihood of new OCS prescriptions by 42%, and that the number of new OCS prescriptions was also reduced (incidence rate 0.82). A very large national study in Japan [82] in severe allergic asthmatic patients reported that omalizumab reduced the dose of OCS from 11.5 mg/day to 5.5 mg/day after 16 weeks and 2.0 mg/day after 52 weeks. A Canadian national study [62] on severe allergic asthma reported that, after 1 year of treatment with omalizumab, the dose of OCS was reduced from 6.3 mg/day to 3.1 mg/day, and that 70.8% of patients either suspended or reduced the dose of OCS by >40%.

In a large long-term study [70], omalizumab reduced the dose of OCS from 10 mg/day to 2 mg at 3 years and then 0 mg/day at 5 years in patients with severe allergic asthma. In a small study, including patients with severe persistent allergic asthma receiving omalizumab for 9 years, only 12.5% of subjects were still under OCS treatment at the end of the observation period [64]. Another small long-term study [81] reported that omalizumab allowed a complete interruption of OCS in 73.3% of severe asthmatic patients. In the remaining patients, the OCS dose was reduced from 22.5 mg/day to 1.83 mg/day.

Additionally, several real-world reports from very large studies are available for omalizumab [66,75,79,83]. A very large study [83] on severe asthmatic patients from a national Czech Republic registry reported that 1 year of treatment with omalizumab decreased the OCS dose from 10.2 mg/day to 5 mg/day, and that 41.1% of patients discontinued OCS therapy. Another very large study [79] in different severe asthma phenotypes showed that after the first year of treatment with omalizumab, 90.4% of patients discontinued the OCS therapy. A study [66] from paper-based and electronic medical records of patients with severe persistent allergic asthma carried out in UK indicated that 1 year of treatment with omalizumab decreased by 1.61 mg/day the dose of OCS. Another investigation [75] performed in severe uncontrolled asthmatic patients showed that omalizumab reduced the OCS courses from 73.8 to 49.2 per 100 patient-years.

In a large study [73] carried out in patients with severe persistent allergic asthma, the OCS dose decreased by 35.5% in the year of treatment with omalizumab, from 21.4 mg/day to 15.9 md/day. In total, 48.5% of patients stopped OCS during the year of observation, and 64% stopped or reduced the dose of OCS dose by ≥20%. Another large study [76] reported that after 16 weeks of treatment, omalizumab reduced by 42.9% the percentage of uncontrolled severe asthmatic patients requiring OCS.

A long-term study [63] showed that after more than 2 years of treatment with omalizumab, 41.9% of patients who had severely difficult to treat asthma were completely weaned off OCS treatment, and that the dose of OCS was reduced from 25.8 mg/day to 6.0 mg/day. The authors also reported that there was a 49% reduction in the number of steroid courses per patient per annum [63]. In uncontrolled severe persistent allergic asthmatic patients with frequent exacerbations observed for 1 year, omalizumab reduced the dose of OCS from 10.6 mg/day to 6.2 mg/day [72].

In a within-person repeated-measures matched cohort [61], omalizumab provided a numerical reduction in the proportion of OCS use after 1 year of treatment from 49.0% to 42.9% in severe asthmatic patients.

In a small study [71] on severe persistent allergic asthma, omalizumab reduced by 77.8% the OCS courses after 1 year of treatment. In another small study [18], the number of severe allergic asthmatic patients who used OCS decreased by 32.1% after initiation of omalizumab.

The rapid and significant OCS-sparing effect of mepolizumab is supported by a large body of real-world evidence, including national and international studies.

### 3.6. Reslizumab

Only three studies [85,86,87] investigated the impact on OCS use of reslizumab in real-world settings.

A very large study [85] showed that 35.1%, 45.9%, and 53.2% of patients with severe eosinophilic asthma discontinued OCS after 4, 7, and 10 months of treatment with reslizumab, respectively. Among the patients that continued to use OCS, the daily dose decreased from 20.6 mg/day to 15.5 mg/day after reslizumab initiation.

In a large study [87] in severe eosinophilic asthmatic patients, reslizumab permitted complete discontinuation of OCS use in 70.3% of patients after 1 year of treatment.

In a small but long-term study [86], 2 years of treatment with reslizumab permitted a reduction of 50% of the number of severe asthmatic patients requiring OCS, and the reduction in the dose of OCS was from 9.3 mg/day at baseline to 4.50 mg/day, 5.23 mg/day, and 4.12 mg/day after 12 weeks, 1 year, and 2 years of treatment, respectively.

### 3.7. Pearson’s Correlation Analysis

**Figure 2:**
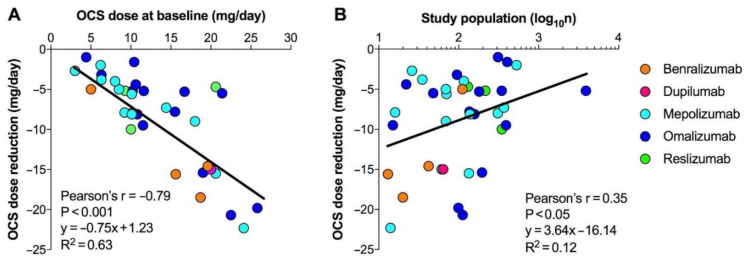
Linear regression and Pearson’s correlation analysis between the reduction in the dose of OCS induced by mAbs and the level of OCS dose at baseline (**A**) or the size of the study population (**B**); the dose of OCS was reported as prednisone equivalent. OCS: oral corticosteroids; mAb: monoclonal antibody.

The Pearson’s correlation analysis indicated that the reduction in the dose of OCS induced by mAbs in asthmatic patients was significantly correlated with the level of OCS dose at baseline (*p* < 0.001) and the size of the population included in the studies (*p* < 0.05, Figure 2). Conversely, no significant correlation was detected between the OCS dose reduction and the duration of the studies (*p* > 0.05, data not shown).

## 4. Discussion

Overall, this systematic review reports that mAbs are effective in eliciting a rapid, significant, and sustained OCS sparing effect in severe asthmatic patients. Such evidence comes from very heterogeneous real-world studies that were very different concerning the number of observed patients and the disease characteristics.

Thus, it is mandatory to adequately interpret these current real-world findings according to the robustness of the studies and the severity and characteristics of the disease. Furthermore, the therapeutic role of OCS and the impact of mAbs to overcome the OCS dependence in asthmatic patients should be considered according to the current GINA recommendations [1]. Specifically, short-course OCS could be administered as an initial controller treatment, with medium-dose maintenance ICS/formoterol at GINA step 4 in those patients presenting severely uncontrolled asthma [1]. However, to control symptoms and minimize future risk in a personalized management of asthma at GINA step 5, maintenance OCS could be added at low dose on high-dose ICS/LABA [1].

In the light of these recommendations, it is also important to assess what is the proportion of adult asthmatic patients who have difficult to treat or severe asthma. According to the last document on the diagnosis and management of difficult to treat and severe asthma, around 24% of asthmatic patients are at GINA step 4-5 and only 17% suffer from difficult to treat asthma; in other words, they are patients at GINA Step 4-5 with poor symptom control [92]. Indeed, patients suffering from difficult to treat asthma are uncontrolled despite medium- or high-dose ICS/LABA with maintenance OCS. Nevertheless, these patients are not necessary “difficult patients” because the difficulty of treating asthma may be related with modifying factors, such as inadequate inhaler technique, poor adherence, smoking habit, and comorbidities [1]. In this case, it is important to highlight that there may be no dependence on OCS, and that the apparent maintenance OCS could be prevented by acting on the above-mentioned modifying factors.

However, a very small amount (3.7%) of patients with difficult to treat asthma really suffer from severe asthma, a condition related to GINA step 4-5, associated with poor symptom control and good adherence and inhaler technique [92]. Indeed, in these patients, it is very possible that the condition of OCS dependence, fortunately, can be overcome by the use of mAbs, due to the real-world findings reported in this systematic review. In fact, the term “refractory asthma” is no longer appropriate for these patients since mAbs are effective in controlling symptoms and reduce or even stop the use of maintenance OCS.

Regardless of the disease characteristics reported for each study, omalizumab seems to be the mAb characterized by the largest amount of evidence resulting from very large and long-term real-world investigations [74,78,82], which, consistently, indicated that this drug may be effective in suspending the use of OCS in more than 50% of severe asthmatic patients, and reducing the daily dose of OCS in more than 50% of the remaining patients. Indeed, the large real-world evidence on omalizumab may be related to the fact that this mAb has been present on the market for a longer time than the other mAbs included in this systematic review.

Interestingly, data on mepolizumab reported from very large and long-term real-world studies [42,50,56] were generally consistent with those obtained for omalizumab with respect to both the percentage of patients that discontinued OCS and the reduction in the daily dose of OCS in those patients that still required maintenance OCS to control the disease. The only very large study [85] on reslizumab also confirmed that mAb therapy is able to halve the percentage of patients requiring maintenance OCS, although reslizumab reduced the daily dose of OCS slightly less than omalizumab and mepolizumab among the patients that continued to need OCS. However, we have to highlight that the daily dose of OCS at baseline in the very large studies on omalizumab [74,78,82] and mepolizumab studies [42,50,56] were generally lower than that of the very large study on reslizumab [85]. Only one large real-world study [34] has been carried out on benralizumab, which permitted OCS consumption to be abolished in all the observed patients. This extremely positive finding may be related to the fact that the baseline OCS daily dose was only 5 mg. Concerning dupilumab, to date, only a small study [40] investigated the OCS-sparing effect in a real-world setting, reporting that 24% of patients completely suspended OCS, and 78% of patients halved the daily OCS dose.

Certainly, the efficacy of the current mAbs in eliciting a certain level of the OCS-sparing effect, and thus overcome the OCS dependence in severe asthmatic patients, should be interpreted not only according to the disease characteristics of the investigated population, but also with the level of OCS dose at baseline. In this respect, the evidence resulting from the Pearson’s correlation analysis indicates that the higher the OCS dose at baseline, the greater the OCS-sparing effect induced by the mAbs. Thus, from a clinical point of view, the real-world evidence suggests that mAbs are more effective in reducing the daily OCS dose in those patients treated with higher levels of OCS at baseline. Moreover, although with a borderline small/medium R^2^ value [93], we found that the size of the real-world studies was negatively and significantly correlated with the OCS-sparing effect, suggesting a so-called “small study effect”, a phenomenon that may lead to overestimation of the efficacy of an active treatment when it is investigated in small populations compared with that observed in larger and more robustly designed studies [94].

Finally, some of the studies [60,67] reported in this systematic review included populations of patients receiving OCS that suffered from moderate, not severe, asthma and that were also treated with mAbs. According to the current recommendations [1], these patients should have been treated neither with OCS nor with mAbs, and thus the results of these studies [60,67] should be interpreted with caution since the patients were overtreated with both OCS and mAbs.

## 5. Conclusions

Real-world studies support the evidence that OCS dependence is a real condition that, however, can be found only in a small number of really severe asthmatic patients. In most patients, the dependence and/or resistance to OCS can be related to modifying factors that, when adequately modulated, may lead to a significant reduction or suspension of OCS maintenance. On the other hand, in severe patients in which OCS resistance is proved by a high daily dose intake, the current pharmacological armamentarium based on the use of mAbs permits reversion of OCS dependence, leading to the suspension of OCS therapy in most patients or a reduction in the daily dose greater than 50% in those subjects affected by very severe forms of asthma. In any case, the overall current real-world findings and data from specific long-term investigations [88,89,90,91] indicate that the level of the OCS-sparing effect and the reversion of OCS dependence is an effect of the mAbs class, regardless of the specific target modulated by each compound. This evidence is in agreement with the position of the current recommendations [1], which do not support one specific mAb against the others for use as an OCS-sparing agent at GINA step 5. Indeed, further studies that aim to assess the impact of different mAbs on specific asthma phenotypes according to the levels of IgE, IL-4, IL-5, and IL-13 may further optimize the efficacy of the current mAbs for the treatment of severe asthma.

## Figures and Tables

**Table 1:** Characteristics of the studies included in the systematic review.

Author, Year, and Reference	mAb	Study Characteristics	Country	Study Duration	Study Population (*n*)	Disease Characteristics	Age (Years)	Male (%)	OCS Use at Baseline (Dose, mg/day) *	Main OCS-Sparing Effects	
										OCS Dose Reduction (mg/day *)	OCS Suspension (%)
Pelaia et al., 2021 [34]	Benralizumab	Real-world, observational, multicenter	Italy	6 months	111	Severe uncontrolled persistent eosinophilic asthma	56.0	64.0	5.0	−5.0	72.2
Scioscia et al., 2021 [35]	Benralizumab	Real-world, prospective, observational, single-center	Italy	6 months	10	Severe eosinophilic asthma	54.0	30	NA	NA	100.0
Menzella et al., 2020 [36]	Benralizumab	Real-world, longitudinal, retrospective, observational, single-center	Italy	6 months	20	Severe refractory asthma	54.0	60.0	18.7	−18.5	95.0
Padilla-Galo et al., 2020 [37]	Benralizumab	Real-world, cross-sectional, multicenter	Italy	6 months	42	Severe refractory eosinophilic asthma	53.6	21.4	19.6	−14.6	NA
Pelaia et al., 2020 [38]	Benralizumab	Real-world, observational, single-center	Italy	6 months	22	Atopic severe uncontrolled persistent eosinophilic asthma	58.5	40.9	25.0	NA	81.8
Pelaia et al., 2019 [39]	Benralizumab	Real-world, observational, single-center	Italy	1 month	13	Severe persistent allergic eosinophilic asthma	56.9	30.8	15.6	−15.6	100.0
Dupin et al., 2020 [40]	Dupilumab	Real-world, retrospective, cohort, multicenter	France	1 year	64	Severe uncontrolled asthma	51.0	46.9	20.0	−15.0	24.0
Enriquez-Rodriguez et al., 2021 [41]	Mepolizumab	Real-world, single-center	Spain	1 year	69	Severe uncontrolled eosinophilic asthma	56.0	28.0	18.0	−9.0	48.0
Thomas et al., 2021 [42]	Mepolizumab	Real-world, observational, multicentre	Australia	1 year	309	Severe eosinophilic asthma	60.0	42.0	10.00	−8.0	34.0
Cameli et al., 2020 [51]	Mepolizumab	Real-world, single-center	Italy	6 months	26	Severe eosinophilic asthma	56.4	65.3	3.0	−2.7	33.3
Crimi et al., 2020 [52]	Mepolizumab	Real-world, single-center	Italy	1 year	31	Severe refractory uncontrolled eosinophilic asthma	52.4	42.0	NA	NA	76.2
Harrison et al., 2020 [50]	Mepolizumab	Real-world, global, prospective, observational, cohort, multicenter	International (UK, Italy, Germany, Canada, Belgium, Spain, USA)	53–56 weeks	368	Severe refractory asthma	53.1	38.0	14.4	−7.3	45.0
Kallieri et al., 2020 [53]	Mepolizumab	Real-world, prospective non-interventional, observational, multicenter	Greece	1 year	70	Severe eosinophilic asthma	55.0	31.4	10.1	−5.6	40.0
Pelaia et al., 2020 [54]	Mepolizumab	Real-world, observational, multicenter	Italy	1 year	88	Severe persistent eosinophilic asthma	54.5	35.2	NA	NA	84.2
Renner et al., 2020 [55]	Mepolizumab	Real-world, prospective, single-center	Austria	5 months	35	Severe eosinophilic asthma and inadequate asthma symptom control	57.4	40.0	6.3	−3.8	NA
Silver et al., 2020 [56]	Mepolizumab	Real-world, retrospective, cohort, from IBM Watson Health MarketScan^®^ Commercial and Encounters Database	USA	2 years (1 year pre-index period and 1 year post-index period)	527	Severe asthma	49.4	49.3	6.2	−2.0	28.4
Sposato et al., 2020 [57]	Mepolizumab	Real-world, observational, multicenter	Italy	6 months	134	Severe asthma	58.3	64.5	NA	NA	45.4
Van Toor et al., 2020 [58]	Mepolizumab	Real-world, retrospective observational, longitudinal, single-center	Netherlands	1 year	78	Severe eosinophilic asthma	54.0	44.0	10.0	NA	50.0
Yilmaz et al., 2020 [43]	Mepolizumab	Real-world, single-center	Turkey	6 months	16	Severe eosinophilic asthma with chronic rhinosinusitis and nasal polyps	48.6	81.0	9.2	−7.9	40.0
Bagnasco et al., 2019 [44]	Mepolizumab	Real-world, single-center	Italy	1 year	138	Severe uncontrolled asthma	58.0	43.5	10.1	−8.1	65.0
Caminati et al., 2019 [45]	Mepolizumab	Real-world, multicenter	Italy	6 months	69	Severe eosinophilic asthma	55.4	39.1	8.5	−5.0	54.3
Schleich et al., 2019 [46]	Mepolizumab	Real-world, prospective, single-center	Belgium	2.5 years	116	Severe eosinophilic asthma	54.0	36	8.0	−4.0	NA
Taillé et al., 2019 [47]	Mepolizumab	Real-world, retrospective, observational, multicenter	France	2 years	134	Severe eosinophilic asthma	58.2	54.8	20.6	−15.5	58.1
Pelaia et al., 2018 [48]	Mepolizumab	Real-world, single-center	Italy	6 months	14	Severe persistent eosinophilic asthma	56.8	21.4	24.1	−22.3	NA
Pertzov et al., 2018 [49]	Mepolizumab	Real-world, single-center	Israel	6 months	61	Severe eosinophilic asthma	57.5	46.6	20.0	−15.0	68.0
Asano et al., 2020 [59]	Omalizumab	Real-world, from the Japanese Pharmaceutical and Medical Devices Agency	Japan	1 year	390	Severe allergic asthma	58.5	41.3	NA	NA	13.3
Frix et al., 2020 [70]	Omalizumab	Real-world, retrospective, observational, single-center	Belgium	5 years	157	Severe allergic asthma	48.0	59.2	11.5	−9.5	NA
Kucharczyk et al., 2020 [78]	Omalizumab	Real-world, long-term, national, observational, retrospective, multicenter	Poland	4 years	989	Severe allergic asthma	46.8	38.0	10.8	−8.1	56.0
Campo et al., 2019 [79]	Omalizumab	Real-world, observational, retrospective, multicenter	Spain	1 year	345	Severe asthma	48.6	33.3	NA	NA	82.0
Kirchnerova et al., 2019 [80]	Omalizumab	Real-world, post-marketing, non-interventional, multicenter, open-label, observational, from international registry	Czech Republic	2 years	112	Severe uncontrolled persistent allergic asthma	44.0	49.3	11.6	−5.2	52.6
Pelaia et al., 2019 [81]	Omalizumab	Real-world, observational, single-center	Italy	5 years	15	Severe allergic asthma	46.6	33.3	22.5	−20.7	73.3
Adachi et al., 2018 [82]	Omalizumab	Real-world, post-marketing, observational, multicenter	Japan	1 year	3893	Severe allergic asthma	59.3	45.3	11.5	−9.5	NA
Hutyrova et al., 2018 [83]	Omalizumab	Real-world, from Czech Anti-IgE Registry	Czech Republic	1 year	310	Severe allergic asthma	44.0	39.7	10.2	−5.2	59.6
Lee et al., 2018 [84]	Omalizumab	Real-world, from retrospective analysis of electrical medical records	Korea	1 year (6 months baseline period and 6 months outcome period)	122	Severe asthma	45.5	40.1	4.4	−1.0	NA
Pilon et al., 2018 [60]	Omalizumab	Real-world, retrospective, multicenter, from electronic medical records	USA	2 years (1 year baseline period and 1 year outcome period)	208	Uncontrolled moderate to severe allergic asthma	41.4	35.1	NA	NA	22.6
Tadrous et al., 2018 [61]	Omalizumab	Real-world, within-person repeated-measures matched cohort, single-center	Canada	2 years (1 year baseline period and 1 year outcome period)	95	Severe uncontrolled asthma	61.1	67.4	NA	NA	12.4
Bhutani et al., 2017 [62]	Omalizumab	Real-world, retrospective, multicenter	Canada	1 year	99	Severe allergic asthma	47.8	68.7	6.3	−3.2	NA
Mansur et al., 2017 [63]	Omalizumab	Real-world, retrospective, registry and case note review, single-center	UK	≥23 months	45	Severe difficult to treat asthma	44.9	19.0	25.8	−19.8	41.9
Menzella et al., 2017 [64]	Omalizumab	Real-world, retrospective, single-center	Italy	9 years	8	Severe persistent allergic asthma	43.0	62.5	NA	NA	85.7
Snelder et al., 2017 [65]	Omalizumab	Real-world, prospective, observational, from national registry	Netherlands	4 months	403	Inadequately controlled severe allergic asthma	47.0	37.0	NA	NA	23.1
Niven et al., 2016 [66]	Omalizumab	Real-world, retrospective, observational, from paper-based and electronic medical records, multicenter	UK	1 year	258	Severe persistent allergic asthma	44.7	34.9	10.4	−1.61	15.8
Chen et al., 2016 [67]	Omalizumab	Real-world, retrospective, population-based, national database, cohort study	Taiwan	1 year	282	Uncontrolled moderate to severe asthma	51.3	NA	NA	NA	37.7
Gibson et al., 2016 [68]	Omalizumab	Real-world, observational, multicenter, from national registry database	Australia	6 months	180	Severe allergic asthma with high prevalence of comorbidities	51.4	46.7	10	NA	4.8
Pereira Barbosa et al., 2015 [69]	Omalizumab	Real-world, observational, national registry	Portugal	2 years	62	Uncontrolled persistent allergic asthma	49.2	30.6	16.7	−5.3	47.5
Gouder et al., 2015 [71]	Omalizumab	Real-world, single-center	Malta	1 year	22	Severe persistent uncontrolled allergic asthma	52.7	64.0	NA	NA	77.8
Sousa et al., 2015 [72]	Omalizumab	Real-world, prospective, observational, multicenter	Portugal	1 year	48	Uncontrolled severe persistent allergic asthma with frequent exacerbations	52.0	33.0	10.6	−4.4	NA
Barnes et al., 2013 [73]	Omalizumab	Real-world, retrospective, observational, multicenter	UK	1 year	136	Severe persistent allergic asthma	41.3	31.6	21.4	−5.5	48.5
Braunstahl et al., 2013 [74]	Omalizumab	Real-world, multinational, non-interventional, observational registry	International	2 years	925	Uncontrolled persistent allergic asthma	46.0	55.7	15.5	−7.8	71.5
Grimaldi-Bensouda et al., 2013 [75]	Omalizumab	Real-world, multicenter	France	NA	767	Severe uncontrolled asthma	51.0	36.8	5.0	NA	33.3
Rottem, 2012 [18]	Omalizumab	Real-world, from insurance database	Israel	10.4 months	33	Severe allergic asthma	50.0	54.5	NA	NA	32.1
Schumann et al., 2011 [76]	Omalizumab	Real-world, multicenter	German	4 months	195	Uncontrolled severe asthma	43.6	40.6	NA	NA	42.9
Molimard et al., 2010 [77]	Omalizumab	Real-world, multicenter	International (French, German)	>4 months	346	Severe persistent allergic asthma	46.1	45.2	19.0	−15.4	59.5
Kavanagh et al., 2021 [87]	Reslizumab	Real-world, observational, single-center	UK	1 year	130	Severe eosinophilic asthma	52.8	38.5	10.0	−10.0	70.3
Wechsler et al., 2021 [85]	Reslizumab	Real-world, retrospective, multicenter, from patient-level data collected via center and panel-based physician chart review	USA	13 months (6 months baseline period and 7 months outcome period)	215	Severe eosinophilic asthma	45.2	56.3	20.6	−4.7	53.2
Ibrahim et al., 2019 [86]	Reslizumab	Real-world, single-center	Ireland	2 years	27	Severe asthma	52.0	38.0	9.3	−5.2	50.0
Bjerrum et al., 2021 [88]	anti-IL-5/IL-5Rα (benralizumab, mepolizumab, reslizumab)	Real-world, retrospective, single-center	Denmark	2 years	81	Severe eosinophilic asthma	55.0	52.0	10.0	−10	62.7
Fong et al., 2021 [89]	Mepolizumab, omalizumab	Real-world, retrospective, single-center	UK	Up to 18 months	167	Severe asthma	56.5	43.3	10.0	Mepolizumab: −5.0; omalizumab: 0.0	NA
Kotisalmi et al., 2020 [90]	Anti-IgE and anti-IL-5/IL-5Rα (benralizumab, mepolizumab, omalizumab, reslizumab)	Real-world, retrospective, single-center	Finland	12.8 months	64	Severe asthma	52.0	38.5	5.9	Anti-IgE: −2.3; anti-IL-5/IL-5Rα: −3.0	NA
Voelker et al., 2020 [91]	anti-IL-5/IL-5Rα (benralizumab, mepolizumab)	Real-world, retrospective, single-center	USA	2 years	63	Severe asthma	54.2	41.0	15.0	−15.0	NA

* prednisone equivalent. mAb: monoclonal antibody; OCS: oral corticosteroid.

## Data Availability

Not applicable.
